# Targeting MicroRNA in Cancer Using Plant-Based Proanthocyanidins

**DOI:** 10.3390/diseases4020021

**Published:** 2016-04-28

**Authors:** Rishipal R. Bansode, Janak R. Khatiwada, Jack N. Losso, Leonard L. Williams

**Affiliations:** 1 Center for Excellence in Post-Harvest Technologies, North Carolina Research Campus, North Carolina Agricultural and Technical State University, Kannapolis, NC 28081, USA; jrkhatiw@ncat.edu (J.R.K.); llw@ncat.edu (L.L.W.); 2 School of Nutrition & Food Sciences, Louisiana State University, Baton Rouge, LA 70803, USA; jlosso@lsu.edu

**Keywords:** proanthocyanidins, microRNA, cancer, polyphenols

## Abstract

Proanthocyanidins are oligomeric flavonoids found in plant sources, most notably in apples, cinnamon, grape skin and cocoa beans. They have been also found in substantial amounts in cranberry, black currant, green tea, black tea and peanut skins. These compounds have been recently investigated for their health benefits. Proanthocyanidins have been demonstrated to have positive effects on various metabolic disorders such as inflammation, obesity, diabetes and insulin resistance. Another upcoming area of research that has gained widespread interest is microRNA (miRNA)-based anticancer therapies. MicroRNAs are short non-coding RNA segments, which plays a crucial role in RNA silencing and post-transcriptional regulation of gene expression. Currently, miRNA based anticancer therapies are being investigated either alone or in combination with current treatment methods. In this review, we summarize the current knowledge and investigate the potential of naturally occurring proanthocyanidins in modulating miRNA expression. We will also assess the strategies and challenges of using this approach as potential cancer therapeutics.

## 1. Introduction

Research related to microRNAs (miRNAs) has gained prominence as a viable cancer therapy [1]. These are short non-encoding RNAs of 20–24 nucleotides that are involved in gene regulation and cell signaling [2].

They have been shown to contain gene-expression regulatory activities, and they can function both as promoters and inhibitors of oncogenesis [3]. The inhibitory activities of miRNA have led researchers to investigate its potential in cancer therapeutics. MicroRNAs are known to achieve this inhibitory role by binding to *cis*-elements in the 3’-untranslated region (UTR) of mRNAs [4]. Following the binding of miRNAs, inhibition occurs by one of three mechanisms: (1) target mRNA site-specific cleavage; (2) repression of target mRNA translation; and (3) mRNA degradation by cytoplasmic processing bodies [5].

MicroRNAs are integral parts of feedback circuits in normal physiology. They play a role in cell differentiation, regulating cellular pathway and exhibiting buffering effects to key biological processes [2,6,7,8,9,10,11,12]. On the other hand, deregulation of miRNA results in enhanced cellular plasticity, dedifferentiation and increased translation of oncogenes [13,14,15].

Over 1400 human miRNAs have been identified, and many of these miRNAs are found to be strongly conserved across species [16]. It is estimated that more than 60% of all mRNAs are regulated by miRNAs via posttrancriptional mode [17]. It is for this reason that recent research has been directed towards understanding these miRNAs in diverse biological processes, including in a variety of diseases.

## 2. Biogenesis of miRNA

**Figure 1:**
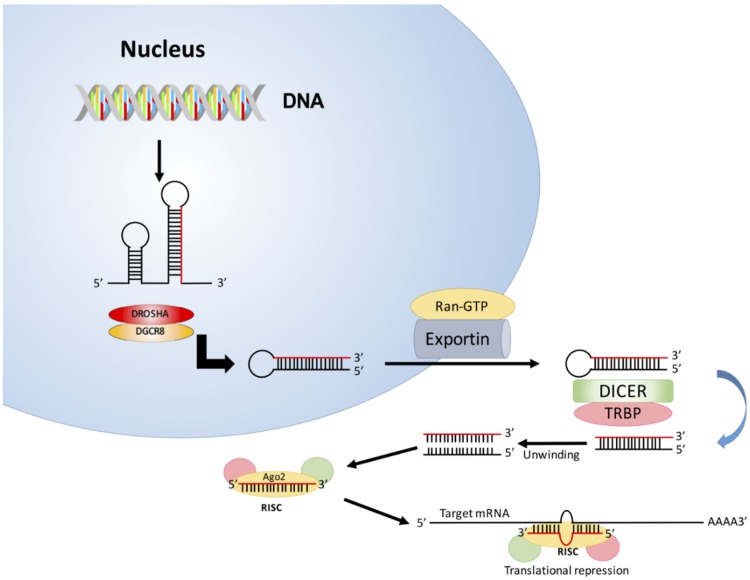
The biogenesis of microRNA.

The canonical pathway of miRNA processing includes transcription by RNA polymerase II to form a primary miRNA (pri-miRNA) transcript with 5′-end caps and 3′-end poly-A tails (Figure 1). This transcript consists of more than one hairpin structure. It is subsequently cleaved by the nuclease, Drosha and its cofactor DiGeorge syndrome critical region 8 (DGCR8), thus generating a precursor miRNA (pre-miRNA). This pre-miRNA is transported from the nucleus to the cytoplasm by exportin-5 (Exp5) using RanGTP as a cofactor. It undergoes further processing in the cytoplasm by RNase III Dicer in a complex with trans-activation response RNA-binding protein (TRBP) to generate approximately 22-nucleotide duplex mature miRNA. Finally, the miRNA duplex unwinds and the functional strand of mature miRNA complexes with Argonaute (Ago2), forming an RNA-induced silencing complex (RISC). This RISC complex directs silencing via sequence-specific mRNAs target cleavage, translational repression and mRNA deadenylation [18].

## 3. Mechanism of Action of miRNAs

**Figure 2:**
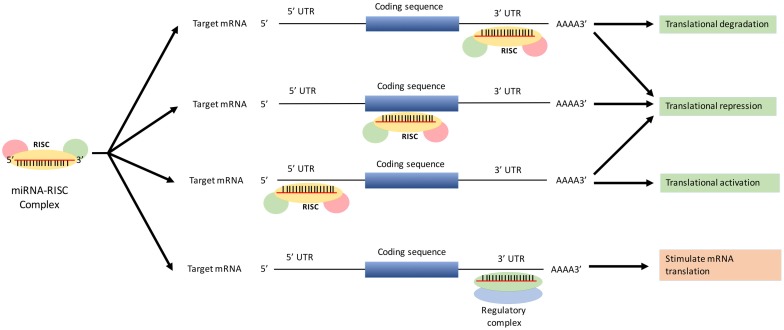
Function of miRNA in translational repression or activation of mRNAs.

The main mechanism with which miRNA effects protein-coding genes is by interaction with the 3′-UTR of target mRNA, subsequently leading to mRNA degradation or translation repression [19]. The interaction of miRNA can also occur at the 5′-untranslated region (5′-UTR) of the protein coding sequence, thereby affecting translation repression or activation of target proteins [20,21]. Translation repression can also occur when miRNA targets the coding sequence of the target protein [22]. Alternately, miRNAs can up-regulate the translation of target genes by interacting with regulatory complexes [23]. The function of miRNAs is illustrated in Figure 2.

## 4. Implication of miRNAs in Cancer

Recent studies have identified deregulation miRNAs in various tumors (Table 1). These miRNAs have been determined to be located in the proximity of the breakpoints, regions of amplification or loss of heterozygocity [24]. Alternately, they can lead to carcinogenesis by altered miRNA expression due to defective miRNA processing, post transcriptional regulation, mutation and epigenetic changes [11,25,26,27]. Their main function in tumorigenesis is to regulate oncogenes or tumor-suppressor genes. These miRNAs that are involved in tumorigenesis are known as oncomirs. They can act as oncogenes by down-regulating genes involved in cell differentiation and apoptosis, or can act as tumor suppressors by down-regulating oncogenes [28]. For example, miR-21 is widely expressed in various types of cancers, and it hinders the activity of tumor-suppressor genes [29,30].

The miR-17-92 cluster (miR-17, miR-18a, miR-19a, miR-19b-1, miR-20a and miR-92-1) also known as oncomiR-1, is aberrantly expressed in various solid tumors [52]. In humans, miR 17-92 is over-expressed in several hematopoietic malignancies and solid tumors [53]. The sequences are highly conserved across species and also within the miR 17-92 family [54]. The expression of miR 17-92 has been studied in animals and human cancer and has been found to show pleiotropic functions [55,56]. Under both normal and malignant transformations, miR 17-92 stimulates proliferation, inhibit differentiation, initiate angiogenesis and promote cell survival.

Members of the let-7 family, mir-15a and mir-16-1, are well-known tumor suppressors that can affect proto-oncogenes like RAS, HMGA2, MYC and IMP-1 [57,58,59,60]. Generally, these miRNAs are down-regulated in most cancers. The oncogenic property of miR 17-92 mainly results in elevated c-myc expression. Other modes of action in carcinogenesis are through the modulation of cell signaling, cell adhesion, hypoxia-related genes and angiogenesis [61,62,63,64].

## 5. Dietary Phytochemical and Chemoprevention

Phytochemicals are important constituents of fruits, vegetables and legumes. These naturally occurring bioactive compounds represent a rich source of micronutrients in the human diet [65]. Epidemiological studies have shown that regular consumption of fruits and vegetables reduce the risk of cancer, metabolic syndrome and age-related diseases [66]. Phytochemicals such as isothiocyanates are known to prevent cancer by affecting cellular mechanisms [67]. A majority of these compounds exhibit antioxidant properties and can regulate carcinogenesis by scavenging reactive oxygen species (ROS) and inhibiting other oxidative stress [68]. Other molecular pathways with which phytochemicals inhibit carcinogenesis are by their anti-inflammatory and pro-apoptotic activity [69]. Recent studies have highlighted that the mechanism of chemoprevention also involves modulating miRNAs that regulate cellular pathways associated with inflammation or apoptosis [70].

## 6. Classification of Polyphenols

Polyphenols represent the most abundant phytochemicals in the human diet. Depending on their chemical structure, they are classified into several groups. Most polyphenols occur in nature as conjugates with sugar or organic acids [71]. The chemical structure consists of a benzene ring attached to one or more hydroxyl groups. They can also form polymers as in the case of flavonoids [72]. Polyphenols and their metabolites can regulate cell functionality by binding to target proteins or by affecting histone modification [73,74,75]. The role of polyphenols in regulating miRNA is not clearly understood. However, recent studies show that polyphenols may be involved in miRNA modulations by multiple mechanisms. Polyphenols can directly bind to miRNAs posttranscriptionally, and they can affect their functionality [76]. They can also bind to mRNAs and proteins, thereby affecting the miRNA biogenesis [72]. The latter involves inhibition of cell signaling by either phosphorylating or dephosphorylating proteins involved in miRNA maturation such as the Dicer complex [77]. Polyphenols can also inhibit transcription factors that bind to the promoter regions of miRNAs [78].

## 7. Structure of Proanthocyanidins

**Figure 3:**
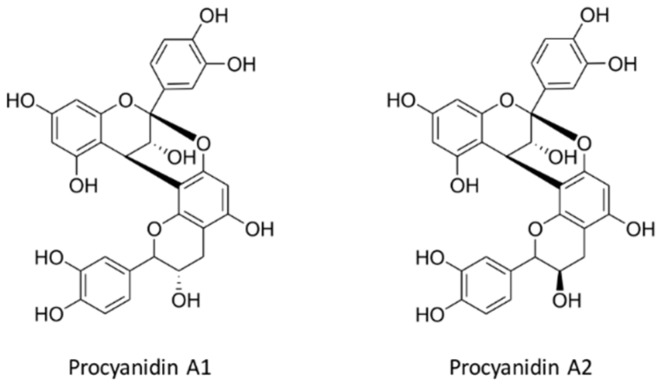

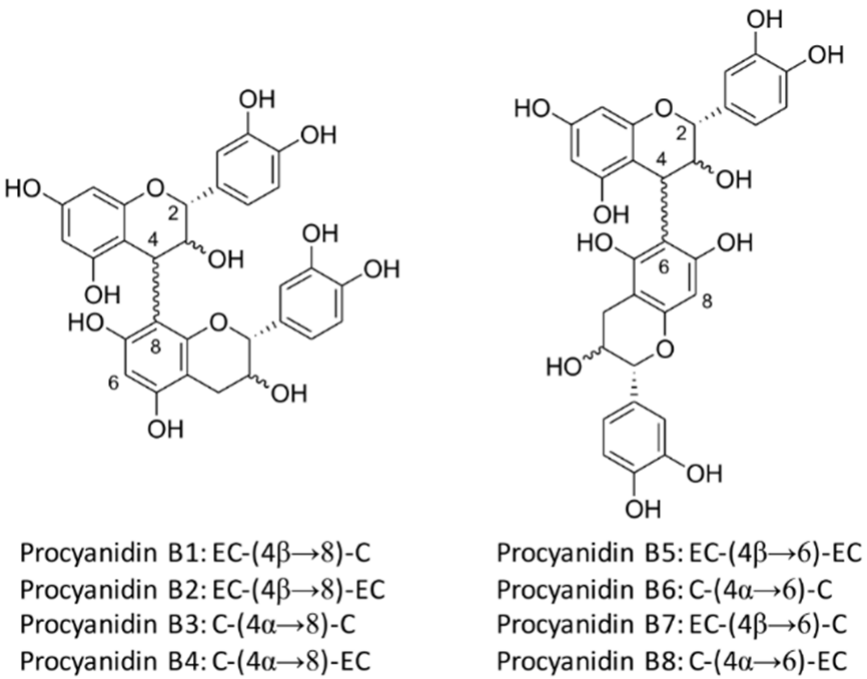
Structure of Type-A and Type-B proanthocyanidins.

Proanthocyanidins are condensed tannins that are the most abundant phytochemicals in nature. Among these, the group that mainly contains (epi)-catechins is known as procyanidins. These procyanidins exist in numerous stereo-chemical isomeric structures (Figure 3). Proanthocyanidins containing (epi)-afzelechin or (epi)-gallocatechin are known as prodelphinidins [79]. They can be broadly differentiated into two subgroups with linkages of catechin or epicatechin at C4-C8 and/or C2-O7 (Type A), or C4-C8 and/or C4-C6 (Type B). They can form complexes with multiple catechins and epicatechins, and with their gallic acid esters to form polymers. The type of polymers are classified as monomers, dimers, trimers, tetramers or oligomers (>4 polymers) [80]. Proanthocyanidins are found in various foods which include their most common B-type polymers. However, some A-type procyanidins predominantly occur in cranberries, plums, avocados, peanuts and cinnamon [79].

Proanthocyanidins like most other flavonoids, exists in glycosylated forms [81]. The type of glycosylation and its associated sugar moiety greatly influence its absorption [82]. The glycosylated proanthocyanidins are stable during digestion and resist acid hydrolysis [83]. They are catabolized by colonic microflora into metabolites that are absorbed into the circulatory system [84]. The metabolites then reach target tissues and undergo glucuronidation, methylation and sulfation [85]. Some studies also reported the bioavailability of unconjugated B-type procyanidins in plasma reaching a peak concentration within two hours of ingestion [86,87,88,89,90].

## 8. Proanthocyanidins Influence Regulation of microRNAs *in Vitro*

Research related to the benefits of proanthocyanidins in the regulation of miRNA is relatively new and is being actively investigated. Recent studies have shown that they can modulate miRNAs that play an important role in cancer, glucose and lipid homeostasis [91,92,93]. Current studies establishing the role of procyanidins in miRNAs regulation are presented in the Table 2. One study involved cranberry extract treatment to esophaegeal adenocarcinoma (EAC) cell lines and its precursor Barrett’s esophagus (BE). This study found that EAC treated with cranberry procyanidin rich extract (CPE) induced miR-410 and miR-520d-5p and reduced miR-202, miR-516a-3p and miR-586 levels [91]. They showed that these five miRNAs are linked to the regulation of target genes that are involved in tumor suppression (p53 and p16), oncogenesis (Rb and Erb8) and inflammation (NFkB). The overall treatment affected 26 genes that play key role in angiogenesis, T-cell activation and apoptosis [91].

Proanthocyanidins extracts from grape seed (GSPE) and cocoa (CPE) differentially expressed miR-30b *, miR-197, miR-532-3p and miR-1224-3p in HepG2 hepatoma cells [93]. This study suggested that the degree of polymerization, botanical origin and growth conditions could affect miRNA regulation of proanthocyanidins. In another study using GSPE in HepG2 cells, miR-122 and miR-33a levels were reduced [76]. These two miRNAs are known to mediate dyslipedimia and insulin resistance [94]. miR-33a/b regulate cholesterol homeostasis and fatty acid β-oxidation, while miR-122 play a vital role in liver homeostasis [95,96,97]. Because miR-33a/b occurs in the intronic region of the sterol response element proteins (SREBF2 and SREBF1), the authors further investigated if the host gene expression would affect the intronic miRNA levels [98]. Their results showed that host gene SREBP2 expression was unaltered while miRNA expression of miR-33 was modulated.

The authors using ^1^H-NMR spectroscopy reported that EGCG and resveratrol could bind to miRNA and thus influence its target genes. This study directly implicates the role of polyphenols at a posttranscriptional level. This outcome inferred that polyphenols can directly bind to miRNAs and can inhibit their posttranscriptional regulation [99].

## 9. Animal Model Studies Establish the Role of Proanthocyanidins in miRNA Regulation

The health-promoting effects of grape proanthocyanidins have been previously studied using animal models [100,101,102,103,104]. Procyanidins are known to exert beneficial effects by regulating lipids homeostasis and reducing atherogenic risk [96]. Studies involving ApoE knockout mice fed with proanthocyanidins for two weeks at a dosage equivalent to human intake of 300 mg/day showed modulation of a total of 55 miRNAs in the livers of Apoe −/− compared to C57BL/6 mice [105]. The highest upregulation was for mmu-miR-133b, a miRNA involved in cell differentiation [106]. Procyanidins exposure at a concentration of 25 mg/kg of bodyweight showed significant downregulation of miR-1249, miR-483, and miR-30c-1 * and upregulation of miR-3544 in pancreatic islets [107]. Proanthocyanidins are also shown to inhibit AsPC-1 pancreatic cancer cell growth and migration by upregulating let-7a [108].

Grape seed proanthocyanidins (GSP) were also shown to suppress miR-106b in melanoma cells (A375, Hs294t) by arresting G1-phase and increasing p21/WAF1/Cip1 protein levels and affecting its downstream signaling by inhibiting pRb, E2F1 and E2F2 [109]. They also corroborated this finding *in vivo* following A375 cells tumor xenograft in nude mice that showed administration of GSP in the diet reduced tumor growth and suppressed miR-106b expression in the tumors [109].

## 10. Conclusions

There is a growing amount of evidence that dietary polyphenols are important micronutrients that can play a role in preventing degenerative diseases. Fruits-and-vegetables-based proanthocyanidins are one of the most abundant phytochemicals. They could serve as a tool to benefit human health. Although studies have been undertaken to establish the role of proanthocyanidins in various cancers using *in vitro* and *in vivo* methods, large scale epidemiological data is lacking. Unlike pharmacological drugs, polyphenols are complex compounds and their chemical and biochemical characteristics are dependent on source and cultivar. Clear evidence of their mechanistic function in regulating cellular responses is still unknown. It is hypothecated that polyphenols could modulate miRNA by directly binding it or interacting with key effector proteins involved in miRNA biogenesis. They can also affect miRNA by indirectly regulating its target genes or by modifying host gene expression as some miRNAs are present in intronic regions of the genes. Given the complexity of their structures and their role in affecting multiple cellular pathways simultaneously, attributing their beneficial effects to a distinctive molecular pathway is challenging. Despite these limitations, recent studies have clearly established their role in cancer-prevention. On a similar level, miRNA’s role in human epigenetics is just being investigated. There are formidable clinical implications of using miRNAs as an effective chemotherapy. These, combined with advances in nutritional diets designed to address specific disease conditions, will serve to improve the fight against cancer.

## Figures and Tables

**Table 1:** Overview of key microRNAs associated with cancer.

MicroRNA	Up- (↑)/Down- (↓) regulation	Cancer type	References
miR-21	↑	Lung cancer	[31]
let-7 family	↓	Lung cancer cells	[32]
miR-125b, mir-145	↓	Breast cancer	[33]
miR-21, mir-155	↑		
miR-27a	↑	Breast cancer	[34,35]
miR-143, mir-145	↓	Colorectal cancer	[36]
miR-106a, mir-21	↑	Colorectal cancer	[37]
miR-15, mir-16	↓	Chronic lymphocytic leukemia (CLL)	[38]
let-7b, let-7c	↓	Acute myeloid leukemia (AML)	[39,40]
miR-24	↑		
miR-29a	↑	B-cell chronic lymphocytic leukemia (CLL)	[41]
miR-155	↑		[42]
miR-125b	↑	B-cell acute lymphocytic leukemia (ALL)	[43]
miR-19	↑	T-cell acute lymphocytic leukemia (ALL)	[44]
miR-21	↑	pre-B malignant lymphoid-like phenotype in mice	[45]
miR-122	↓	Liver cancer	[46]
miR-221, miR-222, miR-21	↑	Hepatocellular carcinoma (HCC)	[47]
miR-103, miR-107	↑	Pancreatic cancer	[48]
miR-141	↑	Prostate cancer	[49]
miR-221	↑	Ovarian cancer	[50]
miR-21, let-7 family	↓		
miR-17-92 cluster	↑	Prostate cancer cells	[51]

**Table 2:** List of miRNAs regulated by proanthocyanidins.

MicroRNA	Up- (↑)/Down- (↓) regulation	Source	Experimental Model	References
miR-30b *	↓	GSPE ^1^, CPE ^2^	HepG2 cells	[93]
miR-1224-3p, miR-197, miR-532-3p	↑			
miR-33a, miR-122	↓	GSPE	Rats, HepG2 cells	[76,99,110]
miR-410, miR-520d-5p	↑	Cranberry-PE ^3^	JHAD1 and OE33 cells	[91]
miR-202, miR-516a-3p, miR-586	↓			
let-7a	↑	GSPE	AsPC-1 cells	[108]
let-7c-2 *, miR-125a-3p, miR-146b, miR-190, miR-190b, miR-196a, miR-196b, miR-197, miR-291b-3p, miR-292, miR-294, miR-297a, miR-29a *, miR-302a, miR-302b, miR-302c, miR-302d, miR-30c-1 *, miR-335-3p, miR-374 *, miR-450a-3p, miR-450b-5p, miR-455 *, miR-464, miR-7b, miR-469, miR-483 *, miR-487b, miR-505, miR-539, miR-542-3p, miR-551b, miR-669a, miR-676, miR-698, miR-7b *, miR-881	↑	GSPE	Apoe^−/−^ mice	[105]
let-7b *, miR-1, miR-106a, miR-133a, miR-133b, miR-17, miR-181a, miR-191 *, miR-200c, miR-291b-5p, miR-296-5p, miR-324-5p, miR-374, miR-486, miR-671-5p, miR-801, miR-878-3p, miR-99b	↓			
miR-3544	↑	GSPE	Pancreatic islets of Wistar rats	[107]
miR-1249, miR-483, miR-30c1 *	↓			
miR-106b	↓	GSPE	A375 cells tumor xenograft in nude mice	[109]

^1^ GSPE: Grape seed proanthocyanidins extract; ^2^ CPE: Cocoa proanthocyanidins extract; ^3^ Cranberry-PE: Cranberry proanthocyanidins extract; * miRNA originated from the opposite arm of the precursor miRNA

## References

[1] Cheng C.J., Bahal R., Babar I.A., Pincus Z., Barrera F., Liu C., Svoronos A., Braddock D.T., Glazer P.M., Engelman D.M. (2015). MicroRNA silencing for cancer therapy targeted to the tumour microenvironment. Nature.

[2] Jansson M.D., Lund A.H. (2012). MicroRNA and cancer. Mol. Oncol..

[3] Shukla S., Meeran S.M., Katiyar S.K. (2014). Epigenetic regulation by selected dietary phytochemicals in cancer chemoprevention. Cancer Lett..

[4] Lynn F.C. (2009). Meta-regulation: MicroRNA regulation of glucose and lipid metabolism. Trends Endocrinol. MeTable.

[5] Carthew R.W., Sontheimer E.J. (2009). Origins and mechanisms of miRNAs and siRNAs. Cell.

[6] Herranz H., Cohen S.M. (2010). MicroRNAs and gene regulatory networks: Managing the impact of noise in biological systems. Genes Dev..

[7] Hornstein E., Shomron N. (2006). Canalization of development by microRNAs. Nat. Genet..

[8] Levine E., McHale P., Levine H. (2007). Small regulatory RNAs may sharpen spatial expression patterns. PLoS Comput. Biol..

[9] Reinhart B.J., Slack F.J., Basson M., Pasquinelli A.E., Bettinger J.C., Rougvie A.E., Horvitz H.R., Ruvkun G. (2000). The 21-nucleotide *let*-7 RNA regulates developmental timing in *Caenorhabditis elegans*. Nature.

[10] Yi R., Poy M.N., Stoffel M., Fuchs E. (2008). A skin microRNA promotes differentiation by repressing ‘stemness’. Nature.

[11] Cordes K.R., Sheehy N.T., White M.P., Berry E.C., Morton S.U., Muth A.N., Lee T.H., Miano J.M., Ivey K.N., Srivastava D. (2009). miR-145 and miR-143 regulate smooth muscle cell fate and plasticity. Nature.

[12] Lim L.P., Lau N.C., Garrett-Engele P., Grimson A., Schelter J.M., Castle J., Bartel D.P., Linsley P.S., Johnson J.M. (2005). Microarray analysis shows that some microRNAs downregulate large numbers of target mRNAs. Nature.

[13] Kumar M.S., Lu J., Mercer K.L., Golub T.R., Jacks T. (2007). Impaired microRNA processing enhances cellular transformation and tumorigenesis. Nat. Genet..

[14] Peter M.E. (2009). Let-7 and miR-200 microRNAs: Guardians against pluripotency and cancer progression. Cell Cycle.

[15] Palmero E.I., de Campos S.G., Campos M., de Souza N.C., Guerreiro I.D., Carvalho A.L., Marques M.M. (2011). Mechanisms and role of microRNA deregulation in cancer onset and progression. Genet. Mol. Biol..

[16] Griffiths-Jones S. (2010). Mirbase: MicroRNA sequences and annotation. Curr Protoc Bioinformatics.

[17] Lewis B.P., Burge C.B., Bartel D.P. (2005). Conserved seed pairing, often flanked by adenosines, indicates that thousands of human genes are microRNA targets. Cell.

[18] Winter J., Jung S., Keller S., Gregory R.I., Diederichs S. (2009). Many roads to maturity: MicroRNA biogenesis pathways and their regulation. Nat. Cell Biol..

[19] Ling H., Fabbri M., Calin G.A. (2013). MicroRNAs and other non-coding RNAs as targets for anticancer drug development. Nat. Rev. Drug Discov..

[20] Lytle J.R., Yario T.A., Steitz J.A. (2007). Target mRNAs are repressed as efficiently by microRNA-binding sites in the 5′ UTR as in the 3′ UTR. Proc. Natl. Acad. Sci. USA.

[21] Eiring A.M., Harb J.G., Neviani P., Garton C., Oaks J.J., Spizzo R., Liu S., Schwind S., Santhanam R., Hickey C.J. (2010). miR-328 functions as an RNA decoy to modulate hnRNP E2 regulation of mRNA translation in leukemic blasts. Cell.

[22] Tay Y., Zhang J., Thomson A.M., Lim B., Rigoutsos I. (2008). MicroRNAs to Nanog, Oct4 and Sox2 coding regions modulate embryonic stem cell differentiation. Nature.

[23] Vasudevan S., Tong Y., Steitz J.A. (2007). Switching from repression to activation: MicroRNAs can up-regulate translation. Science.

[24] Budhu A., Ji J., Wang X.W. (2010). The clinical potential of microRNAs. J. Hematol. Oncol..

[25] Deng S., Calin G.A., Croce C.M., Coukos G., Zhang L. (2008). Mechanisms of microRNA deregulation in human cancer. Cell Cycle.

[26] Schmittgen T.D. (2008). Regulation of microRNA processing in development, differentiation and cancer. J. Cell Mol. Med..

[27] Thomson J.M., Newman M., Parker J.S., Morin-Kensicki E.M., Wright T., Hammond S.M. (2006). Extensive post-transcriptional regulation of microRNAs and its implications for cancer. Genes Dev..

[28] Zhang B., Pan X., Cobb G.P., Anderson T.A. (2007). MicroRNAs as oncogenes and tumor suppressors. Dev. Biol..

[29] Yang C.H., Pfeffer S.R., Sims M., Yue J., Wang Y., Linga V.G., Paulus E., Davidoff A.M., Pfeffer L.M. (2015). The oncogenic microRNA-21 inhibits the tumor suppressive activity of FBXO11 to promote tumorigenesis. J. Biol. Chem..

[30] Zhou X., Wang X., Huang Z., Wang J., Zhu W., Shu Y., Liu P. (2014). Prognostic value of miR-21 in various cancers: An updating meta-analysis. PLoS ONE.

[31] Seike M., Goto A., Okano T., Bowman E.D., Schetter A.J., Horikawa I., Mathe E.A., Jen J., Yang P., Sugimura H. (2009). miR-21 is an EGFR-regulated anti-apoptotic factor in lung cancer in never-smokers. Proc. Natl. Acad. Sci. USA.

[32] Takamizawa J., Konishi H., Yanagisawa K., Tomida S., Osada H., Endoh H., Harano T., Yatabe Y., Nagino M., Nimura Y. (2004). Reduced expression of the let-7 microRNAs in human lung cancers in association with shortened postoperative survival. Cancer Res..

[33] Iorio M.V., Ferracin M., Liu C.G., Veronese A., Spizzo R., Sabbioni S., Magri E., Pedriali M., Fabbri M., Campiglio M. (2005). MicroRNA gene expression deregulation in human breast cancer. Cancer Res..

[34] Mertens-Talcott S.U., Chintharlapalli S., Li X., Safe S. (2007). The oncogenic microRNA-27a targets genes that regulate specificity protein transcription factors and the G2-M checkpoint in MDA-MB-231 breast cancer cells. Cancer Res..

[35] Li X., Mertens-Talcott S.U., Zhang S., Kim K., Ball J., Safe S. (2010). MicroRNA-27a indirectly regulates estrogen receptor α expression and hormone responsiveness in MCF-7 breast cancer cells. Endocrinology.

[36] Michael M.Z., SM O.C., van Holst Pellekaan N.G., Young G.P., James R.J. (2003). Reduced accumulation of specific microRNAs in colorectal neoplasia. Mol. Cancer Res..

[37] Link A., Balaguer F., Shen Y., Nagasaka T., Lozano J.J., Boland C.R., Goel A. (2010). Fecal microRNAs as novel biomarkers for colon cancer screening. Cancer Epidemiol. Biomarkers. Prev..

[38] Calin G.A., Dumitru C.D., Shimizu M., Bichi R., Zupo S., Noch E., Aldler H., Rattan S., Keating M., Rai K. (2002). Frequent deletions and down-regulation of micro- RNA genes miR15 and miR16 at 13q14 in chronic lymphocytic leukemia. Proc. Natl. Acad. Sci. USA.

[39] Jongen-Lavrencic M., Sun S.M., Dijkstra M.K., Valk P.J., Lowenberg B. (2008). MicroRNA expression profiling in relation to the genetic heterogeneity of acute myeloid leukemia. Blood.

[40] Zaidi S.K., Dowdy C.R., van Wijnen A.J., Lian J.B., Raza A., Stein J.L., Croce C.M., Stein G.S. (2009). Altered Runx1 subnuclear targeting enhances myeloid cell proliferation and blocks differentiation by activating a miR-24/MKP-7/MAPK network. Cancer Res..

[41] Pekarsky Y., Croce C.M. (2010). Is miR-29 an oncogene or tumor suppressor in CLL?. Oncotarget.

[42] Wang M., Tan L.P., Dijkstra M.K., van Lom K., Robertus J.L., Harms G., Blokzijl T., Kooistra K., van T'veer M B., Rosati S. (2008). miRNA analysis in B-cell chronic lymphocytic leukaemia: Proliferation centres characterized by low miR-150 and high BIC/miR-155 expression. J. Pathol..

[43] Bousquet M., Harris M.H., Zhou B., Lodish H.F. (2010). MicroRNA miR-125B causes leukemia. Proc. Natl. Acad. Sci. USA.

[44] Mavrakis K.J., Wolfe A.L., Oricchio E., Palomero T., de Keersmaecker K., McJunkin K., Zuber J., James T., Khan A.A., Leslie C.S. (2010). Genome-wide RNA-mediated interference screen identifies miR-19 targets in Notch-induced T-cell acute lymphoblastic leukaemia. Nat. Cell Biol..

[45] Medina P.P., Nolde M., Slack F.J. (2010). Oncomir addiction in an *in vivo* model of microRNA-21-induced pre-B-cell lymphoma. Nature.

[46] Tsai W.C., Hsu P.W., Lai T.C., Chau G.Y., Lin C.W., Chen C.M., Lin C.D., Liao Y.L., Wang J.L., Chau Y.P. (2009). MicroRNA-122, a tumor suppressor microRNA that regulates intrahepatic metastasis of hepatocellular carcinoma. Hepatology.

[47] Yoon S.O., Chun S.M., Han E.H., Choi J., Jang S.J., Koh S.A., Hwang S., Yu E. (2011). Deregulated expression of microRNA-221 with the potential for prognostic biomarkers in surgically resected hepatocellular carcinoma. Hum. Pathol..

[48] Roldo C., Missiaglia E., Hagan J.P., Falconi M., Capelli P., Bersani S., Calin G.A., Volinia S., Liu C.G., Scarpa A. (2006). MicroRNA expression abnormalities in pancreatic endocrine and acinar tumors are associated with distinctive pathologic features and clinical behavior. J. Clin. Oncol..

[49] Mitchell P.S., Parkin R.K., Kroh E.M., Fritz B.R., Wyman S.K., Pogosova-Agadjanyan E.L., Peterson A., Noteboom J., O’Briant K.C., Allen A. (2008). Circulating microRNAs as stable blood-based markers for cancer detection. Proc. Natl. Acad. Sci. USA.

[50] Dahiya N., Sherman-Baust C.A., Wang T.L., Davidson B., Shih I.M., Zhang Y., Wood W., Becker K.G., Morin P.J. (2008). MicroRNA expression and identification of putative miRNA targets in ovarian cancer. PLoS ONE.

[51] Zhou P., Ma L., Zhou J., Jiang M., Rao E., Zhao Y., Guo F. (2016). miR-17–92 plays an oncogenic role and conveys chemo-resistance to cisplatin in human prostate cancer cells. Int. J. Oncol..

[52] Mogilyansky E., Rigoutsos I. (2013). The miR-17/92 cluster: A comprehensive update on its genomics, genetics, functions and increasingly important and numerous roles in health and disease. Cell Death Differ..

[53] Ota A., Tagawa H., KaRNAn S., Tsuzuki S., Karpas A., Kira S., Yoshida Y., Seto M. (2004). Identification and characterization of a novel gene, *C13orf25*, as a target for 13q31-q32 amplification in malignant lymphoma. Cancer Res..

[54] Olive V., Jiang I., He L. (2010). miR-17–92, a cluster of miRNAs in the midst of the cancer network. Int. J. Biochem. Cell Biol..

[55] He L., Thomson J.M., Hemann M.T., Hernando-Monge E., Mu D., Goodson S., Powers S., Cordon-Cardo C., Lowe S.W., Hannon G.J. (2005). A microRNA polycistron as a potential human oncogene. Nature.

[56] Olive V., Li Q., He L. (2013). miR-17–92: A polycistronic oncomir with pleiotropic functions. Immunol. Rev..

[57] Johnson S.M., Grosshans H., Shingara J., Byrom M., Jarvis R., Cheng A., Labourier E., Reinert K.L., Brown D., Slack F.J. (2005). Ras is regulated by the let-7 microRNA family. Cell.

[58] Lee Y.S., Dutta A. (2007). The tumor suppressor microRNA let-7 represses the HMGA2 oncogene. Genes Dev..

[59] Sampson V.B., Rong N.H., Han J., Yang Q., Aris V., Soteropoulos P., Petrelli N.J., Dunn S.P., Krueger L.J. (2007). MicroRNA let-7A down-regulates MYC and reverts MYC-induced growth in Burkitt lymphoma cells. Cancer Res..

[60] Boyerinas B., Park S.M., Shomron N., Hedegaard M.M., Vinther J., Andersen J.S., Feig C., Xu J., Burge C.B., Peter M.E. (2008). Identification of let-7-regulated oncofetal genes. Cancer Res..

[61] Mei Q., Li X., Guo M., Fu X., Han W. (2014). The miRNA network: Micro-regulator of cell signaling in cancer. Expert Rev. Anticancer. Ther..

[62] Valastyan S., Weinberg R.A. (2011). Roles for microRNAs in the regulation of cell adhesion molecules. J. Cell Sci..

[63] Favaro E., Ramachandran A., McCormick R., Gee H., Blancher C., Crosby M., Devlin C., Blick C., Buffa F., Li J.L. (2010). MicroRNA-210 regulates mitochondrial free radical response to hypoxia and krebs cycle in cancer cells by targeting iron sulfur cluster protein ISCU. PLoS ONE.

[64] Suarez Y., Sessa W.C. (2009). MicroRNAs as novel regulators of angiogenesis. Circ. Res..

[65] Blumberg J.B., Camesano T.A., Cassidy A., Kris-Etherton P., Howell A., Manach C., Ostertag L.M., Sies H., Skulas-Ray A., Vita J.A. (2013). Cranberries and their bioactive constituents in human health. Adv. Nutr..

[66] Greiner A.K., Papineni R.V., Umar S. (2014). Chemoprevention in gastrointestinal physiology and disease. Natural products and microbiome. Am. J. Physiol. Gastrointest. Liver Physiol..

[67] Russo M., Spagnuolo C., Tedesco I., Russo G.L. (2010). Phytochemicals in cancer prevention and therapy: Truth or dare?. Toxins (Basel).

[68] Su Z.Y., Shu L., Khor T.O., Lee J.H., Fuentes F., Kong A.N. (2013). A perspective on dietary phytochemicals and cancer chemoprevention: Oxidative stress, Nrf2, and epigenomics. Top. Curr. Chem..

[69] Tan A.C., Konczak I., Sze D.M., Ramzan I. (2011). Molecular pathways for cancer chemoprevention by dietary phytochemicals. Nutr. Cancer.

[70] Milenkovic D., Jude B., Morand C. (2013). MiRNA as molecular target of polyphenols underlying their biological effects. Free Radic. Biol. Med..

[71] Clifford M.N.B., Brown J.E., Andersen Ø.M., Markham K.R. (2006). Dietary Flavonoids and Health-Broadening the Perspective.

[72] Fraga C.G., Galleano M., Verstraeten S.V., Oteiza P.I. (2010). Basic biochemical mechanisms behind the health benefits of polyphenols. Mol. Aspects Med..

[73] Blade C., Arola L., Salvado M.J. (2010). Hypolipidemic effects of proanthocyanidins and their underlying biochemical and molecular mechanisms. Mol. Nutr. Food Res..

[74] Malireddy S., Kotha S.R., Secor J.D., Gurney T.O., Abbott J.L., Maulik G., Maddipati K.R., Parinandi N.L. (2012). Phytochemical antioxidants modulate mammalian cellular epigenome: Implications in health and disease. Antioxid. Redox Signal..

[75] Hardy T.M., Tollefsbol T.O. (2011). Epigenetic diet: Impact on the epigenome and cancer. Epigenomics.

[76] Baselga-Escudero L., Arola-ARNAl A., Pascual-Serrano A., Ribas-Latre A., Casanova E., Salvado M.J., Arola L., Blade C. (2013). Chronic administration of proanthocyanidins or docosahexaenoic acid reverses the increase of miR-33a and miR-122 in dyslipidemic obese rats. PLoS ONE.

[77] Chendrimada T.P., Gregory R.I., Kumaraswamy E., Norman J., Cooch N., Nishikura K., Shiekhattar R. (2005). TRBP recruits the Dicer complex to Ago2 for microRNA processing and gene silencing. Nature.

[78] George J., Singh M., Srivastava A.K., Bhui K., Roy P., Chaturvedi P.K., Shukla Y. (2011). Resveratrol and black tea polyphenol combination synergistically suppress mouse skin tumors growth by inhibition of activated mapks and p53. PLoS ONE.

[79] Gu L., Kelm M.A., Hammerstone J.F., Beecher G., Holden J., Haytowitz D., Prior R.L. (2003). Screening of foods containing proanthocyanidins and their structural characterization using LC-MS/MS and thiolytic degradation. J. Agric. Food Chem..

[80] Zhang L., Wang Y., Li D., Ho C.T., Li J., Wan X. (2016). The absorption, distribution, metabolism and excretion of procyanidins. Food Funct..

[81] Manach C., Scalbert A., Morand C., Remesy C., Jimenez L. (2004). Polyphenols: Food sources and bioavailability. Am. J. Clin. Nutr..

[82] Thilakarathna S.H., Rupasinghe H.P. (2013). Flavonoid bioavailability and attempts for bioavailability enhancement. Nutrients.

[83] Gee J.M., DuPont M.S., Rhodes M.J., Johnson I.T. (1998). Quercetin glucosides interact with the intestinal glucose transport pathway. Free Radic. Biol. Med..

[84] Tabasco R., Sanchez-Patan F., Monagas M., Bartolome B., Victoria Moreno-Arribas M., Pelaez C., Requena T. (2011). Effect of grape polyphenols on lactic acid bacteria and bifidobacteria growth: Resistance and metabolism. Food Microbiol..

[85] Selma M.V., Espin J.C., Tomas-Barberan F.A. (2009). Interaction between phenolics and gut microbiota: Role in human health. J. Agric. Food Chem..

[86] Baba S., Osakabe N., Natsume M., Terao J. (2002). Absorption and urinary excretion of procyanidin B2 [epicatechin-(4β-8)-epicatechin] in rats. Free Radic. Biol. Med..

[87] Holt R.R., Lazarus S.A., Sullards M.C., Zhu Q.Y., Schramm D.D., Hammerstone J.F., Fraga C.G., Schmitz H.H., Keen C.L. (2002). Procyanidin dimer B2 [epicatechin-(4β-8)-epicatechin] in human plasma after the consumption of a flavanol-rich cocoa. Am. J. Clin. Nutr..

[88] Sano A., Yamakoshi J., Tokutake S., Tobe K., Kubota Y., Kikuchi M. (2003). Procyanidin B1 is detected in human serum after intake of proanthocyanidin-rich grape seed extract. Biosci. Biotechnol. Biochem..

[89] Shoji T., Masumoto S., Moriichi N., Akiyama H., Kanda T., Ohtake Y., Goda Y. (2006). Apple procyanidin oligomers absorption in rats after oral administration: Analysis of procyanidins in plasma using the porter method and high-performance liquid chromatography/tandem mass spectrometry. J. Agric. Food Chem..

[90] Tsang C., Auger C., Mullen W., Bornet A., Rouanet J.M., Crozier A., Teissedre P.L. (2005). The absorption, metabolism and excretion of flavan-3-ols and procyanidins following the ingestion of a grape seed extract by rats. Br. J. Nutr..

[91] Kresty L.A., Clarke J., Ezell K., Exum A., Howell A.B., Guettouche T. (2011). MicroRNA alterations in barrett's esophagus, esophageal adenocarcinoma, and esophageal adenocarcinoma cell lines following cranberry extract treatment: Insights for chemoprevention. J. Carcinog..

[92] Kong A.N., Zhang C., Su Z.Y. (2013). Targeting epigenetics for cancer prevention by dietary cancer preventive compounds—The case of miRNA. Cancer Prev. Res. (Phila.).

[93] Arola-Arnal A., Blade C. (2011). Proanthocyanidins modulate microRNA expression in human HEPG2 cells. PLoS ONE.

[94] Deiuliis J.A. (2016). MicroRNAs as regulators of metabolic disease: Pathophysiologic significance and emerging role as biomarkers and therapeutics. Int. J. Obes. (Lond.).

[95] Gerin I., Clerbaux L.A., Haumont O., Lanthier N., Das A.K., Burant C.F., Leclercq I.A., MacDougald O.A., Bommer G.T. (2010). Expression of miR-33 from an SREBP2 intron inhibits cholesterol export and fatty acid oxidation. J. Biol. Chem..

[96] Del Bas J.M., Crescenti A., Arola-Arnal A., Oms-Oliu G., Arola L., Caimari A. (2015). Intake of grape procyanidins during gestation and lactation impairs reverse cholesterol transport and increases atherogenic risk indexes in adult offspring. J. Nutr. Biochem..

[97] Tsai W.C., Hsu S.D., Hsu C.S., Lai T.C., Chen S.J., Shen R., Huang Y., Chen H.C., Lee C.H., Tsai T.F. (2012). MicroRNA-122 plays a critical role in liver homeostasis and hepatocarcinogenesis. J. Clin. Invest..

[98] Horie T., Ono K., Horiguchi M., Nishi H., Nakamura T., Nagao K., Kinoshita M., Kuwabara Y., Marusawa H., Iwanaga Y. (2010). MicroRNA-33 encoded by an intron of sterol regulatory element-binding protein 2 (SREBP2) regulates hdl *in vivo*. Proc. Natl. Acad. Sci. USA.

[99] Baselga-Escudero L., Blade C., Ribas-Latre A., Casanova E., Suarez M., Torres J.L., Salvado M.J., Arola L., Arola-Arnal A. (2014). Resveratrol and EGCG bind directly and distinctively to miR-33a and miR-122 and modulate divergently their levels in hepatic cells. Nuclei. Acids Res..

[100] Vinson J.A., Mandarano M.A., Shuta D.L., Bagchi M., Bagchi D. (2002). Beneficial effects of a novel IH636 grape seed proanthocyanidin extract and a niacin-bound chromium in a hamster atherosclerosis model. Mol. Cell Biochem..

[101] Tong H., Song X., Sun X., Sun G., Du F. (2011). Immunomodulatory and antitumor activities of grape seed proanthocyanidins. J. Agric. Food Chem..

[102] Ding Y., Dai X., Jiang Y., Zhang Z., Bao L., Li Y., Zhang F., Ma X., Cai X., Jing L. (2013). Grape seed proanthocyanidin extracts alleviate oxidative stress and er stress in skeletal muscle of low-dose streptozotocin- and high-carbohydrate/high-fat diet-induced diabetic rats. Mol. Nutr. Food Res..

[103] Ding Y., Dai X., Jiang Y., Zhang Z., Li Y. (2014). Functional and morphological effects of grape seed proanthocyanidins on peripheral neuropathy in rats with type 2 diabetes mellitus. Phytother. Res..

[104] Katiyar S.K. (2016). Dietary proanthocyanidins inhibit uv radiation-induced skin tumor development through functional activation of the immune system. Mol. Nutr. Food Res..

[105] Milenkovic D., Deval C., Gouranton E., Landrier J.F., Scalbert A., Morand C., Mazur A. (2012). Modulation of miRNA expression by dietary polyphenols in apoE deficient mice: A new mechanism of the action of polyphenols. PLoS ONE.

[106] Feng Y., Niu L.L., Wei W., Zhang W.Y., Li X.Y., Cao J.H., Zhao S.H. (2013). A feedback circuit between miR-133 and the ERK1/2 pathway involving an exquisite mechanism for regulating myoblast proliferation and differentiation. Cell Death Dis..

[107] Castell-Auvi A., Cedo L., Movassat J., Portha B., Sanchez-Cabo F., Pallares V., Blay M., Pinent M., Ardevol A. (2013). Procyanidins modulate microRNA expression in pancreatic islets. J. Agric. Food Chem..

[108] Ma J., Fang B., Ma C., Pang H., Zeng F., Xia J. (2015). Proanthocyanidins inhibit pancreatic cancer AsPC-1 cell growth and migration through up-regulation of let-7a. Nan Fang Yi Ke Da Xue Xue Bao.

[109] Prasad R., Katiyar S.K. (2014). Down-regulation of miRNA-106b inhibits growth of melanoma cells by promoting G1-phase cell cycle arrest and reactivation of p21/WAF1/Cip1 protein. Oncotarget.

[110] Baselga-Escudero L., Blade C., Ribas-Latre A., Casanova E., Salvado M.J., Arola L., Arola-Arnal A. (2012). Grape seed proanthocyanidins repress the hepatic lipid regulators miR-33 and miR-122 in rats. Mol. Nutr. Food Res..

